# Acid β-glucosidase (*GBA1*) gene mutational spectrum and clinical phenotypes in patients with gaucher disease: seven novel mutations in a multicenter retrospective cohort study from upper Egypt

**DOI:** 10.1186/s40348-025-00200-5

**Published:** 2025-10-01

**Authors:** Mervat A. M. Youssef, Solaf M. Elsayed, Khalid I. Elsayh, Sherin A. Taha, Hala S.M. Abdelmotogaly, Mostafa M. Embaby

**Affiliations:** 1 Pediatric Hematology Unit, Children’s Hospital, Faculty of Medicine, Assiut University, Assiut, Egypt; 2 Medical Genetic Department, Faculty of Medicine, Ain Shams University, Cairo, Egypt; 3 Pediatric Department, Faculty of Medicine, Suez University, Suez, Egypt; 4 Pediatric department, Faculty of Medicine, New Valley University, Kharga, Egypt

**Keywords:** Gaucher disease, Genotype/phenotype, Correlation, New variants

## Abstract

**Background:**

This study aimed to identify *GBA1* variants in Egyptian Gaucher disease (GD) patients residing in a region with high consanguinity and to correlate these genotypes with their clinical phenotypes.

**Methodology:**

This descriptive study included 68 Egyptian patients diagnosed with GD. Diagnosis relied upon reduced β-glucocerebrosidase activity measured by tandem mass spectrometry from dried blood spots and confirmed by *GBA1* single-gene sequencing. Clinical and laboratory information were gathered from patient records, and neurological evaluations were conducted by a neurologist.

**Results:**

Thirty patients (44.1%) were classified as type 1 GD, three (4.4%) as type 2 GD, and 35 patients (51.5%) as type 3 GD. Variant analysis of the 136 alleles identified 19 different variants. The most prevalent mutant allele was c.1448T > C p.(Leu483Pro) (50.7%). Seven novel variants were documented: five homozygous missense variants, including c.263 C > T p.(Met88Thr), c.1331 A > G p.(Asp444Gly), c.1409 C > T p.(Ser470Phe), c.907 C > G p.(Leu303Val), c.1574G > A p.(Gly525Asp), two heterozygous missense variants: c.380 C > G p.(Ala127Gly) and c.453 + 2T > C. All carriers of these novel variants were phenotypically classified as type 1 GD. Genotype–phenotype correlations confirmed that the c.1226 A > G p.(Asn409Ser) variant was confined to type 1 GD, whereas c.1448T > C p.(Leu483Pro) was associated with types 2 and 3 GD.

**Conclusion:**

Variant analysis of 136 alleles identified 19 *GBA1* variants, including seven novel variants. These findings enhance genotype–phenotype correlations, provide genetic counseling, and enable customized molecular analyses for families at risk.

## Introduction

Gaucher disease (GD) is one of the most common autosomal recessive lysosomal storage diseases caused by variants in the acid β-glucosidase (*GBA1)* gene, which encodes the glucocerebrosidase enzyme [1]. Deficiency of enzymatic activity leads to progressive glucocerebrosidase accumulation, primarily in the mononuclear phagocyte system, within macrophages [2]. GD is a multisystemic disease with heterogeneous phenotypes; however, it is clinically classified into three types based on age of onset, neurological involvement, and disease progression [3]. Type 1 (non-neuronopathic) is the most common subtype and presents with early onset of symptoms, hematological involvement, hepatosplenomegaly, and skeletal manifestations. Type 2 GD (acute neuronopathic) is the most serious subtype and presents in early infancy with rapid brain deterioration. Type 3 GD (chronic neuronopathic) is a subacute form where clinical expressions start in childhood and adolescence [3, 4].

The *GBA1* gene, located on chromosome 1q22, has a highly homologous pseudogene sequence. Thus, variant detection is not easy, and amplification primers are required to discriminate between the functional gene and the pseudogene. More than 721 different *GBA1* gene variants have been reported in GD [5]. Numerous variants have been associated with specific clinical characteristics; however, genotype-phenotype correlation is characterized by variable expression and appearance in different individuals and ethnic groups. The most frequent variants in GD patients are c.1226 A > G p.(Asn409Ser) and c.1448T > C p.(Leu483Pro) [6]. The most frequent variant recognized in Romanian patients is p.(Asn409Ser) [7], while c.1226 A > G p.(Asn409Ser) and c.1448T > C p.(Leu483Pro) are common in Spain [6]. In Ashkenazi Jewish patients, the p.(Asn409Ser) variant, along with c.84dupG (84GG) and IVS2 + 1G > A, represent approximately 96% of variants, whereas in non-Jewish patients these three account for 50–60% of variants [8]. In Caucasian patients, 60–70% of variants are c.1226 A > G p.(Asn409Ser), c.1448T > C p.(Leu483Pro), and c.1342G > C p.(Asp448His) [8].

Thus, c.1226 A > G p.(Asn409Ser) variant has not been associated with neurological involvement and is usually found in type 1 GD. The c.1448T > C p.(Leu483Pro) variant is associated with neurological manifestations and typically allied with type 2 or type 3 GD, even in the heterozygous form [6, 8]. Moreover, the c.680 A > G p.(Asn227Ser) and c.1180G > T p.(Val394Leu) alleles are linked to type 3 GD, accompanied by myoclonic epilepsy [9]. This study was designed to identify variants in Egyptian GD patients living in an area of high consanguinity and correlate their genotypes with clinical characteristics.

## Methodology

### Patients

This descriptive study was carried out in the Hematology Unit of Assiut University Children’s Hospital in Egypt and the Genetics Department of Ain Shams University. The study involved 68 Egyptian patients with GD and was approved by the Ethics and Research Committee of the Faculty of Medicine, Assiut University (approval number: 04-2023-300227). All participants signed consent forms. All recruited patients were diagnosed with GD based on reduced β-glucocerebrosidase activity and was confirmed by *GBA1* genotyping.

### Data collection

All enrolled patients underwent a thorough medical history assessment, including the duration of their illness and relevant family history. Clinical and laboratory information at presentation was gathered from patient records, including the age of presentation. All patients underwent a comprehensive clinical examination, including weight and height assessment, evaluation of liver and spleen size (both clinically and by ultrasound), and an ophthalmological examination. Neurological evaluations were conducted by a neurologist. GD type was categorized clinically by the treating physicians. Type 1 GD (chronic non-neuronopathic subtype) was defined by the absence of neurological symptoms throughout the disease course. Type 2 GD (acute infantile neuronopathic) and type 3 GD (chronic neuronopathic) were based on the age at which neurological signs and symptoms first manifested, and the rate of progression. Type 2 GD presents with neurological symptoms that appear within the first few months of life and progress rapidly, often resulting in severe degeneration and mortality in infancy or early childhood. Type 3 GD is typically associated with a variety of symptoms, including visceral involvement, oculomotor apraxia, and, in some instances, bulbar signs such as stridor, strabismus, feeding difficulties, epilepsy, ataxia, or dementia [10].

### β-Glucosidase enzyme assay

The β-Glucosidase enzyme assay was conducted using tandem mass spectrometry on dried blood spots. Enzyme activity levels were considered reduced if below the cut-off value of 1.5 µmol/L/h.

### Single-gene sequencing (GBA1 gene)

DNA was extracted from dried blood spot samples. Polymerase Chain Reaction (PCR) amplification and sequencing were performed for all coding exons and flanking intronic regions of the *GBA1* gene. The sequences were aligned to the reference sequence NM_000157.3 (ENST00000368373) at the international medical laboratory Archimed Life. Detected variants were described according to the Human Genome Variation Society (HGVS) recommendations for the description of sequence variants (2016 update: Hum Mutat. 2016 Jun;37(6):564-9) [11].

## Results

Sixty-eight patients (45 males and 23 females) participated in this study. Patient ages ranged from 5 months to 58 years, with a median age of 21 years. The age at diagnosis ranged from 4 months to 57 years. All patients exhibited reduced or absent β-glucocerebrosidase activity, accompanied by clinical manifestations consistent with GD. The demographic data of the patients are presented in Table 1. Thirty patients (44.1%) were classified as type 1 GD, with a mean age of 8.93 ± 13.66 years. A positive family history was noted in 21 patients (70%), and 23 patients (77.3%) reported parental consanguinity. Type 2 GD was identified in three patients, all of whom unfortunately died before reaching the age of 2 years. Type 3 GD was the most prevalent form, identified in 35 patients (51.5%), with a consanguinity rate of 28 patients (80%).

**Table 1:** Demographic, clinical, and biochemical data of our cohort

	Type 1	Type 2	Type 3
*n* (%)	30 (44.1)	3 (4.4)	35 (51.5)
Gender, *n* (%)			
Male Female	20 (66.7) 10 (33.3)	2 (66.7) 1 (33.3)	23 (65.7) 12 (36.3)
Age at diagnosis (years)			
Mean ± SD Median (min-max)	8.93 ± 13.66 4.35 (0.9–57)	0.9 ± 0.1 0.9 (0.8-1)	2.3 ± 1.8 1.85 (0.5–10.3)
Current age (years)			
Mean ± SD Median (min-max)	11.11 ± 12.38 8.15 (2.3–58)	--- ---	6.7 ± 5.8 5.1 (1.4–17.1)
Presenting Manifestations	Hepatosplenomegaly, Bone involvement, Thrombocytopenia, Pancytopenia, Leucopenia, Anemia	Hepatosplenomegaly, Progressive CNs involvement in the form of strabismus, bulbar signs, spasticity refractory Epilepsy	Hepatosplenomegaly, Cytopenia, Cognitive problems, Developmental delays, Eye movement disorders, Poor coordination, Seizures
Positive Family history, *n* (%)	21 (70.0)	1 (33.3)	17 (48.5)
Consanguinity	23 (77.3)	2 (66.7)	28 (80)
Dead, *n* (%)	0 (0.0)	3 (100.0)	2 (5.7)
Age of death (years)		&	€
	--- ---	1.6 1.8 1.4	12.8 8.5
β-Glucosidase activity			
Mean (SD) Median (Range)	0.37 ± 0.30 0.3 (0.1–1.2)	0.23 ± 0.13 0.2 (0.1–0.5)	0.24 ± 0.17 0.2 (0.0–0.8)
Lyso-GL-1 Mean (SD) Median (Range)	1289.26 ± 1241.24 877 (450–5504)	1230.58 ± 763.86 994 (261–2584)	1196.12 ± 968.18 1000 (450–5485)

&: 3 patients died; €: 2 patients died

### Pathogenic variants and types detected in the studied group

A total of 136 alleles from 68 patients across 47 independent families were analyzed. The most prevalent variant was c.1448T > C p.(Leu483Pro), accounting for 50.7% (*n* = 69) of all mutant alleles. In contrast, the c.1226 A > G p.(Asn409Ser) variant represented 7.35% (*n* = 10) of the identified mutant alleles. Among the identified variants, 128 (94%) were missense, 2 (1.5%) were nonsense variants, and 6 (4.4%) were recombinant alleles (Table 2).

**Table 2:** Variants and allele frequencies in the studied group (68 patients, 136 alleles, from 47 independent families). All variants were described according to NM_000157.4 (*GBA* transcript) and NP_000148.2 (*GBA* protein)

Transcript	Protein	Molecular Consequence	Classification	Allele Frequency *n* (%)
c.1448T > C	p.Leu483Pro	Missense	Pathogenic	69 (50.7)
c.1226 A > G	p.Asn409Ser	Missense	Pathogenic/likely pathogenic	10 (7.35)
*c.263 C > T	p.Met88Thr	Missense	Pathogenic	8 (5.88)
*c.1331 A > G	p.Asp444Gly	Missense	Pathogenic	8 (5.88)
*c.1409 C > T	p.Ser470Phe	Missense	Pathogenic	6 (4.4)
c.710 A > C	p.Lys237Thr	Missense	Pathogenic	5 (3.67)
c.1604G > A	p.Arg535His	Missense	Pathogenic/Likely pathogenic	5 (3.67)
c.847T > C	p.Tyr283His	Missense	Likely pathogenic	4 (2.9)
1265_1319del55+1448T > C;1483G > C;1497G > C		Recombinant allele	Pathogenic	4 (2.9)
c.1342G > C	p.Asp448His	Missense	Pathogenic/Likely pathogenic	2 (1.47)
*c.907 C > G	p.Leu303Val	Missense	Pathogenic	2 (1.47)
c.1193G > A	:p.Arg398Gln	Missense	Pathogenic/Likely pathogenic	2 (1.47)
c.475 C > T	p.Arg159Trp	Missense	Pathogenic	2 (1.47)
c.680 A > G	:p.Asn227Ser	Missense	Pathogenic	2 (1.47)
*c.1574G > A	p.Gly525Asp	Missense	Pathogenic	2 (1.47)
*c.380 C > G	p.Ala127Gly	Missense	Pathogenic	1 (0.7)
c.754T > A	p.Phe252lle	Missense	Pathogenic/Likely pathogenic	1 (0.7)
*c.453 + 2T > C	–	Splice Site	Pathogenic	1 (0.7)

*Novel variants

We documented seven novel variants within the cohort (Table 2). The first variant, c.263 C > T p.(Met88Thr), was a homozygous missense variant observed in four patients (5.88%) from a single family (three boys and one girl). The symptoms first appeared in the eldest sibling at the age of five years with anemia and hepatosplenomegaly. Subsequent screening of family members led to the identification of the remaining cases. The second novel variant, c.1331 A > G p.(Asp444Gly), was also a homozygous missense variant found in four patients across two families. Three patients (two boys and one girl) belonged to the same family and were diagnosed at the ages of 10, 7, and 4 years with symptoms, including hepatosplenomegaly and bleeding of varying severity. The eldest sibling suffered from severe bone pain with a history of right femur fracture (Table 1).

The third variant, c.1409 C > T p.(Ser470Phe), was a homozygous missense variant identified in three patients (4.4%) from three different families. All three patients presented with anemia, hepatosplenomegaly, and easy bruising. The fourth, c.907 C > G p.(Leu303Val), and fifth, c.1574G > A p.(Gly525Asp), variants were identified as missense variants in a single patient. The sixth, c.380 C > G, p.(Ala127Gly), was a heterozygous missense variant detected in a 2-year-old girl presenting with hepatosplenomegaly with stunted growth. The seventh, [453 + 2T > C], was a heterozygous missense variant identified in a 23-year-old female with splenomegaly and thrombocytopenia associated with heavy menstrual bleeding. All patients carrying these novel variants displayed low enzyme activity and clinical manifestations consistent with type 1 GD, as none developed neurological signs throughout the disease course (Table 3).

**Table 3:** Genotype-phenotype correlation of the studied group (All variants were described according to NM_000157.4 (*GBA* transcript) and NP_000148.2 (*GBA* protein))

No. of patients	No. of families	Allele 1	Allele 2	No. of Patients/Phenotype			Frequency
				Type I	Type 2	Type 3	
31	# 21	c.1448T > C, p.Leu483Pro	c.1448T > C, p.Leu483Pro	-	3	28	45.58%
4	#3	c.1448T > C, p.Leu483Pro	1265_1319del55+1448T > C;1483G > C;1497G > C			4	5.88%
4	&2	c.1226 A > G, :p.Asn409Ser	c.1226 A > G, :p.Asn409Ser	4			5.88%
4	&1	*c.263 C > T, **p. p.Met88Thr**	*c.263 C > T, **p. p.Met88Thr**	4			5.88%
4	$2	*c.1331 A > G, **p. Asp444Gly**	*c.1331 A > G, **p. Asp444Gly**	4			5.88%
3	&3	*c.1409 C > T, **p.Ser470Phe**	*c.1409 C > T, **p.Ser470Phe**	3			4.4%
1	&1	c.1448T > C, p.Leu483Pro	*c.1574G > A, **p.Gly525Asp**	1			2.9%
2	$2	c.847T > C, p.Tyr283His	c.847T > C, p.Tyr283His	2			2.9%
2	&1	c.1604G > A, p.Arg535His	c.1604G > A, p.Arg535His	2			2.9%
2	&1	c.475 C > T, p.Arg159Trp	c.680 A > G, :p.Asn227Ser	2			2.9%
2	&1	c.710 A > C, 710 A > C, p.Lys237Thr	:c.710 A > C, p.Lys237Thr			2	2.9%
1	1	c.710 A > C, p.Lys237Thr	c.475 C > T, p.Arg159Trp			1	1.47%
1	&1	c.1448T > C, p.Leu483Pro	c.1604G > A, p.Arg535His	1			1.47%
1	1	c.1448T > C, p.Leu483Pro	c.1226 A > G, :p.Asn409Ser	1			1.47%
1	&1	c.1193G > A, p.Arg398Gln	c.1193G > A, p.Arg398Gln	1			1.47%
1	1	*c.907 C > G, **p.Leu303Val**	*c.907 C > G, **:p.Leu303Val**	1			1.47%
1	&1	c.1342G > C, p.Asp448His	c.1342G > C, p.Asp448His	1			1.47%
1	&1	c.1226 A > G, :p.Asn409Ser	*c.453 + 2T > C	1			1.47%
1	&1	c.1448T > C, p.Leu483Pro	*c.380 C > G, **p.Ala127Gly**	1			1.47%
1	&1	c 1342G > C (p Asp448His)	c 1342G > C (p Asp448His)	1			1.47%

*Novel variants; &: positive consanguinity; positive consanguinity in 19 family; #: positive consanguinity in 2 family; $: positive consanguinity in one family

### Phenotype /genotype correlation

#### Homozygous variants

Eleven homozygous *GBA1* gene variants were identified among fifty-five patients (Table 3). The most prevalent variant was c.1448T > C p.(Leu483Pro), which was found in 31 patients (45.58%). Among these, 28 patients were phenotypically classified as type 3 GD (chronic neuronopathic) due to the late onset of neurological symptoms. The remaining three patients exhibited features of type 2 GD (acute neuronopathic), characterized by progressive central nervous system involvement, including strabismus, bulbar signs, and spasticity, leading to early mortality before two years of age (Table 1). The homozygous variant c.710 A > C p.(Lys237Thr) was detected in two patients (2.94%), both phenotypically categorized as type 3 GD. Additionally, nine different homozygous *GBA1* gene variants were observed in 22 patients, all of whom showed no neurological signs throughout the disease course and were thus classified as type 1 GD (Table 3).

#### Compound heterozygous

Thirteen patients were found to have compound heterozygous *GBA1* gene variants. The most frequently observed compound variant was c.1448T > Cp.(Leu483Pro)/1265_1319del55;1448T > C;1483G > C;1497G > C, identified in four patients who exhibited a range of symptoms, including visceral and chronic neurological involvement (type 3 GD). An additional compound heterozygous variant, c.710 A > C p.(Lys237Thr)/c.754T > A p.(Phe252lle), was identified in a single patient whose clinical manifestations were consistent with type 3 GD. The remaining eight patients were phenotypically classified as type 1 GD and carried six different compound heterozygous variants (Table 3).

## Discussion

In this study, we describe the clinical characteristics and *GBA1* variant spectrum of 68 patients with GD from Upper (southern) Egypt, all of whom received GD-specific enzyme replacement therapy, imiglucerase (Cerezyme^®^), at the hematology units of Assiut and Ain Shams University Children’s Hospitals. GD exhibits significant heterogeneity in both clinical manifestations and genetic variation, a phenomenon evident in our patient cohort. Clinically, patients exhibited a wide range of phenotypes associated with GD; 30 patients (44.1%) were classified as type 1, 3 patients (4.4%) as type 2, and 35 patients (51.5%) as type 3. Among those with the most severe manifestations, we identified patients with progressive central nervous system involvement, with fatalities occurring as early as 1.5 years of age. At the other end of the spectrum, we identified a patient with mild disease diagnosed at the age of 57 years after the detection of GD in family members. The former patient was homozygous for c.1448T > C p.(Leu483Pro), whereas the latter was homozygous for c.1226 A > G p.(Asn409Ser), a variant often associated with very mild disease.

Although GD is an autosomal recessive disease, we observed a male predominance with a 2:1 ratio. This is consistent with previous Egyptian studies that reflect cultural behavior in Upper Egypt, where males receive greater medical care and attention [12]. In Upper Egypt, GD is the most diagnosed sphingolipidosis and the second most common lysosomal storage disease after mucopolysaccharidoses. It results from biallelic pathogenic variants in the *GBA1* gene, located on chromosome 1q21, which comprises 11 exons [13]. More than 700 distinct *GBA1* variants have been reported globally, with distribution varying significantly by population [14]. The most common variants worldwide include c.1226 A > G p.(Asn409Ser) (previously N370S); c.1448 T > C p.(Leu483Pro) (previously L444P); c.115 + 1G > A (previously IVS2 + 1G > A); and c.84dupG p.(Leu29AlafsTer18) (previously 84 GG [84-85insG]) [15].

The results of this study show that the most frequent *GBA1* variant was c.1448T > C p.(Leu483Pro) (50.7%), followed by c.1226 A > G p.(Asn409Ser) (7.35%), matching other Egyptian studies that reported the *c.1448T > C* variant in 50–60% of cases [16, 17]. Our findings also align with studies from Turkey, Iran, and the JAPAC region (China, India, Japan, Korea, Malaysia, and the Philippines) [18–22]; however, some populations report lower prevalences ranging between 18.5% and 25% [7, 23, 24]. In European, American, Brazilian, Venezuelan, and Jewish populations, c.1226 A > G p.(Asn409Ser) is the most frequently reported variant [25–28]. Thus, c.1226 A > G variant prevalence declines from west to east, whereas the prevalence of the c.1448T > C p.(Leu483Pro) variant increases in the same direction.

In our study, the pathogenic c.1448T > C p.(Leu483Pro) variant was detected in both homozygous and compound heterozygous forms, with frequencies of 45.8% and 10.3%, respectively. In the homozygous state, this variant was associated with type 2 (3/3; 100%) and type 3 (28/35; 80%) phenotypes. Among patients with type 1 GD, c.1448T > C appeared in a heterozygous combination with c.1226 A > G p.(Asn409Ser), c.1604G > A p.(Arg535His), and c.380 C > G p.( Ala127Gly) in 3 out of 30 cases (10%). These patients require close monitoring due to their subtle course and potential late neurological manifestations.

Genotype–phenotype correlation in GD is not absolute. In our study, several patients homozygous for c.1448T > C were initially diagnosed as type I GD based on early presentations of hepatosplenomegaly and cytopenia without neurological manifestations. However, with long-term follow-up, these patients developed supranuclear gaze palsy and cognitive impairment, prompting reclassification to type 3 GD. This occurred despite ongoing enzyme replacement therapy, which appeared to delay, but did not prevent neurological progression [17]. Conversely, some patients initially classified as type 3 based on neurological involvement were reclassified to type 2 due to rapid clinical deterioration and early mortality. Notably, survival beyond two years is atypical for classical type 2 GD. While the homozygous L444P mutation is traditionally linked to Type 3 Gaucher disease [12], our study shows that, in uncommon instances it could lead to a phenotype resembling Type 2, possibly as a result of unidentified genetic modifiers or hidden variants [29]. Additional genomic and functional investigations are necessary to clarify the mechanisms underlying this unusual severity.

Despite having the neuropathic *GBA1* genotype, Egyptian patients with type 3 GD often exhibit a milder phenotype and improved outcomes with enzyme replacement therapy, suggesting potential modifying genes that enhance prognosis following treatment [30–32]. However, not all patients with neuropathic GD carry the c.1448T > C p.(Leu483Pro) variant. In our study, we identified two known variants associated with type 3 GD. The c.710 A > C p.(Lys237Thr) variant, one of the most common variants globally, was found in two patients in the homozygous state, and in one patient in compound heterozygosity with c.754T > A p.(Phe252lle). The latter is a unique pathogenic variant in Asian populations and has been reported in three neuronopathic GD patients in Morocco [33, 34].

The second GAB1 variant identified in this study was the missense c.1226 A > G p.(Asn409Ser) variant. It was detected in four patients in the homozygous state and in two patients in the heterozygous state, all of whom were diagnosed with GD type 1 without neurological involvement. The frequency of this variant is consistent with previous Egyptian studies, which reported it in 8.7% of 195 patients (34 alleles out of 390) [12]. The c.1226 A > G p.(Asn409Ser) variant is prevalent among Ashkenazi Jews, comprising 75–80% of alleles; however, it is rare or absent in populations such as the Japanese [8, 35, 36]. This variant is considered neuroprotective, being exclusive to type 1 patients. The compound heterozygous variant c.1448T > C p.(Leu483Pro)/c.1226 A > G p.(Asn409Ser) was found in one patient. The two alleles are rarely found together in Egyptian cohorts, except for isolated cases reported by Khalifa et al. [17] and Fateen et al. [12]. In contrast, this genotype is more frequent in Spanish and Portuguese populations [37].

This study detected known *GBA1* homozygous variants: c.847T > C p.(Tyr283His) and c.1604G > A p.(Arg535His), detected in two patients each, and c.1193G > A p.(Arg398Gln) and c.1342G > C p.(Asp448His), detected in one patient each. Additionally, c.475 C > T p.(Arg159Trp) and c.680 A > G p.(Asn227Ser) was detected in two patients as compound heterozygous variants. All these variants were detected in type 1 GD patients and were previously reported in the literature. Furthermore, our study identified two different recombinant alleles in six of our patients (4.4% of total alleles and 8.8% of patients). To date, over 20 recombinant *GBA1* alleles have been identified, with RecNcil and Recdelta55 being most prevalent, either alone or in conjunction with other point variants [38, 39]. RecNcil originates from a cross-over between intron 9 and exon 10, leading to the incorporation of a C.P1 segment into the functional *GBA1* gene, including the missense variants c.1448 T > C p.(L483P), c.1483G > C p.(A495P), and c.1497G > C p.(V499 V). Recdelta55 [c.1265_1319del p.(Leu422fs)] involves a 55-bp deletion in exon 9 of the *GBA1* gene, corresponding to the deleted portion of the pseudogene [40]. Recombinant alleles that include the 55-bp deletion in exon 9 were reported with other point variants or with RecNcil [41, 42].

In our study, the recombinant variant RecNcil (c.1574G > A/RecNcil) was identified in a compound heterozygous state in two patients with type 1 GD. Interestingly, a complex and rare variant resulting from the recombination of Rec1263del55 and RecNcil alleles was identified in four patients (5.88%) with type 3 GD in a compound heterozygous form with c.1448T > C p.(Leu483Pro) [40]. Nevertheless, these patients exhibit phenotypic heterogeneity. The frequency of recombinant alleles (Rec) in our study was lower than previously reported rates, which include 38.1% in Egyptian patients with type 1 GD [16], and 9.5–21% reported in other international studies [23, 43].

Our study identified known *GBA1* variants such as c.847T > C p.(Tyr283His), c.1604G > A p.(Arg535His), each in two patients, and c.1193G > A p.(Arg398Gln), c 1342G > C (p Asp448Hs); each in one patient, all were homozygous. Additionally, c.475 C > T p.(Arg159Trp) and c.680 A > G p.(Asn227Ser) were identified in two patients as compound heterozygous variants. All variants were identified in type 1 GD patients, as previously documented in the literature. In addition, our study revealed seven novel variants in 22% of our cohort: c.263 C > T p.(Met88Thr) and c.1331 A > G p.(Asp444Gly) each in four homozygous patients; c.1409 C > T p.(Ser470Phe) in three patients; and c.907 C > G p.(Leu303Val) in one patient, all in homozygous form. The remaining variants, c.1574G > A p.(Gly525Asp) and c.380 C > G p.(Ala127Gly), were each found in one patient as compound heterozygous variants along with the other known variants. All patients carrying these novel variants exhibited reduced enzyme levels and clinical symptoms characteristic of type 1 GD, without neurological signs during disease progression. None of these variants have been previously reported in PubMed or publicly available databases such as ClinVar, HGMD, or dbSNP.

In addition, a splice-site variant, c.453 + 2T > C in intron 4, was identified and located within the intronic regions. Although its exact protein has not been determined, it may be disease-related due to a splicing alteration. This variant was detected in a 22-year-old female patient with type 1 GD, in heterozygosity with c.1226 A > G p.(Asn409Ser). We found a high rate of consanguinity (77.9%) in our cohort, resulting in the high frequency of homozygous variants (80%). The Middle East, particularly Upper Egypt, has some of the world’s highest consanguinity rates (21–33%), contributing to elevated homozygosity even among non-related couples in rural communities due to the long-standing national traditions [44].

## Conclusion

In conclusion, this study presents the findings of 68 patients with GD from Upper Egypt, covering the entire spectrum of disease phenotypes. Analysis of the 136 mutant alleles identified 19 different variants (including 7 novel variants) and 19 genotypes. The c.1448T > C p.(Leu483Pro) variant was the most prevalent (50.7%), with the homozygous form representing the most common genotype (45.5%). Genotype–phenotype correlations confirmed that the c.1226 A > G p.(Asn409Ser) variant is not associated with the development of neurological complications, whereas the c.1448T > C p.(Leu483Pro) variant was strongly linked to severe neuronopathic phenotypes (types 2 and 3 GD). The distribution of mutant alleles and the frequency of specific genotypes exhibit distinct characteristics that may reflect the ethnic features of our community. Identifying these variants expands the understanding of the global *GBA1* variant landscape, provides insights into the disease’s molecular basis, establishes genotype–phenotype correlations, supports genetic counseling, and performs customized molecular analyses for families at risk.

## Data Availability

No datasets were generated or analysed during the current study.

## Abbreviations

CNS: Central Nervous System
GD: Gaucher Disease
GBA1: Glucosylceramidase Beta 1
HGMD: Human Gene Mutation Database
HGVS: Human Genome Variation Society
PCR: Polymerase Chain Reaction
Rec: Recombinant Allele
RecNcil: Recombinant NciI
